# Association between early life antibiotic use and childhood overweight and obesity: a narrative review

**DOI:** 10.1017/gheg.2018.16

**Published:** 2018-10-24

**Authors:** Uttara Partap, Sophie H. Allcock, Edyth Parker, Deepti Gurdasani, Elizabeth H. Young, Manjinder S. Sandhu

**Affiliations:** 1 Department of Medicine, University of Cambridge, Cambridge, UK; 2 Wellcome Sanger Institute, Hinxton, UK; 3 Department of Veterinary Medicine, University of Cambridge, UK

**Keywords:** Antibiotics, child obesity, child overweight, early life

## Abstract

**Background:**

Recent research implicates antibiotic use as a potential contributor to child obesity risk. In this narrative review, we examine current observational evidence on the relation between antibiotic use in early childhood and subsequent measures of child body mass.

**Methods:**

We searched PubMed, Web of Science and the Cochrane Library to identify studies that assessed antibiotic exposure before 3 years of age and subsequent measures of body mass or risk of overweight or obesity in childhood.

**Results:**

We identified 13 studies published before October 2017, based on a total of 6 81 332 individuals, which examined the relation between early life antibiotic exposure and measures of child body mass. Most studies did not appropriately account for confounding by indication for antibiotic use. Overall, we found no consistent and conclusive evidence of associations between early life antibiotic use and later child body mass [minimum overall adjusted odds ratio (aOR) reported: 1.01, 95% confidence interval (95% CI) 0.98–1.04, *N* = 2 60 556; maximum overall aOR reported: 2.56, 95% CI 1.36–4.79, *N* = 616], with no clinically meaningful increases in weight reported (maximum increase: 1.50 kg at 15 years of age). Notable methodological differences between studies, including variable measures of association and inclusion of confounders, limited more comprehensive interpretations.

**Conclusions:**

Evidence to date is insufficient to indicate that antibiotic use is an important risk factor for child obesity, or leads to clinically important differences in weight. Further comparable studies using routine clinical data may help clarify this association.

## Introduction

Child obesity is a public-health issue of increasing global concern. In 2013, the age-standardised prevalence of overweight and obesity among children and adolescents aged <20 years was estimated to be 23.8% and 22.6% in high-income countries, and 12.9% and 13.4% in low- and middle-income countries, among boys and girls, respectively [1–3]. The same year, over 42 million (7%) children under 5 years of age were estimated to be overweight or obese, with prevalence expected to increase to 11% by 2025 if current trends remain constant [1–3]. Obesity during childhood is a risk factor for short- and long-term adverse outcomes, including type-2 diabetes, hypertension and dyslipidaemia [4]. Given the anticipated burden on individuals, health systems and economies, effective evidence-based strategies to prevent and manage child obesity are essential.

Obesity risk is understood to result from a complex interplay of environmental and genetic factors, including well-studied contributions from diet, physical activity and maternal factors such as smoking and breastfeeding [5–7]. Additionally, increasing evidence has implicated alterations in the intestinal microbiome in obesity development [8, 9]. In particular, antibiotic use in early life has been hypothesised to promote obesity through metabolic dysregulation caused by changes in the intestinal microbial composition [10, 11]. This dysregulation is thought to encompass multiple potential pathways, including increases in microbiota-derived energy, decreased energy requirements due to lowered intestinal defence, changes in hepatic function and altered metabolic signalling [11].

Global antibiotic consumption was 34.8 billion daily defined doses (DDDs) in 2015, a 65% increase compared with 2000 levels [12, 13]. Evidence among children suggests notable but varied prevalence across populations and population subgroups [14–20]. For example, antibiotic use among children up to 2 years of age was estimated to be 1365 prescriptions per 1000 persons in the USA in 2010 [16], and 462 prescriptions per 1000 persons in Sweden in 2012 [19]. In both cases, these were among the highest ratios across all age groups [16, 19]. This widespread use of antibiotics among children, and their hypothesised metabolic effects [11], suggests the need to more clearly determine the potential link between antibiotic use and obesity in this age group.

A number of studies have assessed associations between antibiotic exposure and measures of body mass in children, with inconsistent results regarding the existence and strength of associations [21–33]. A more detailed understanding of these studies is required to better identify and define future research aims and inform public-health intervention. In this narrative review, we aim to synthesise and summarise the current evidence on the relation between early life antibiotic use and measures of child body mass.

## Methods

We searched PubMed, Web of Science and the Cochrane Library in order to identify studies published before October 2017 assessing the relation between early childhood antibiotic use and measures of child body mass. Key words for antibiotics and body mass-related outcomes were searched and combined using Boolean operators as appropriate (online Supplementary Methods).

### Definitions of exposure and outcome

Early childhood antibiotic use was defined as exposure to any pharmaceutical agent with antibacterial action between birth and 3 years of age. This time period was selected as the microbiome is thought to be dynamic and in development until it matures at this age [34], with antibiotic administration during this period hypothesised to have a pronounced impact on the development of the microbiome composition and related metabolic functioning. No restrictions were placed on the nature or frequency of the antibiotic exposure, or on the method of ascertainment of antibiotic exposure.

Given the wide variation in definition of overweight and obesity among children in research and clinical practice [1, 35–37], measures of body mass included any outcome based on absolute or relative weight or body mass index (BMI) between 2 and 18 years of age. This included BMI-for-age, weight-for-height or binary measures such as overweight or obesity based upon any of these indices. Studies examining self-reported or parental-reported outcomes were included. The upper age limit of the outcome was chosen given the interest in examining long-term implications of early life antibiotic use across childhood.

### Inclusion and exclusion criteria

We placed no restrictions on study design, publication date or status in our selection criteria. Only English language publications were reviewed. Studies based on children who were extremely malnourished or with an underlying disease, or in children born low birth weight or small for gestational age, were excluded, given potential differences in their microbiome profile relative to generally healthy, normal weight children [38, 39].

### Literature search and synthesis

**Fig. 1.**
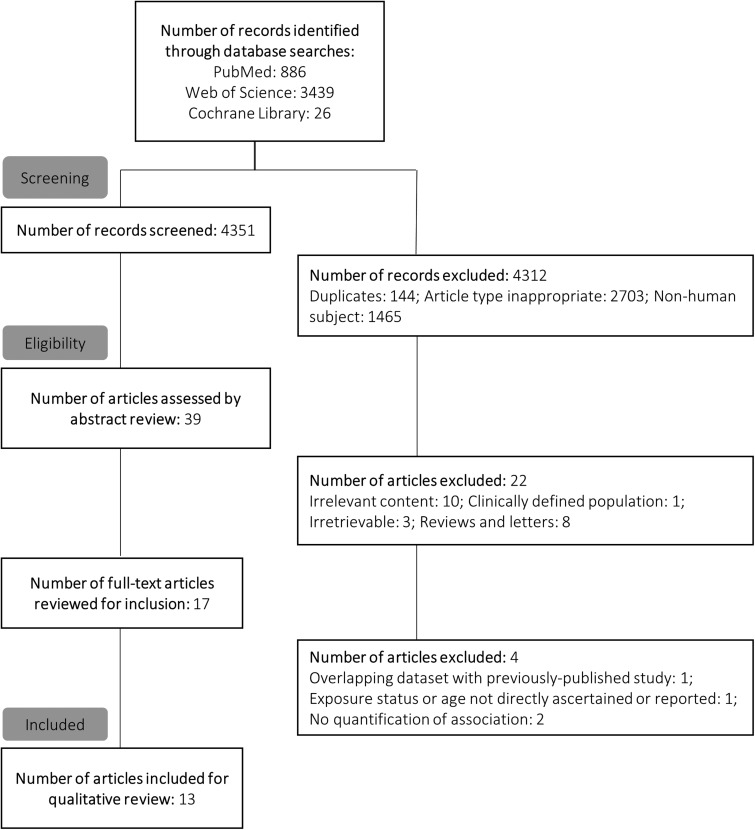
Schematic of literature search and exclusion for final review.

An initial search was performed by one author (EP) to identify studies published before March 2016. This was repeated in October 2017 by a second author (UP) to confirm the initial search results and identify more recently published studies (Fig. 1). Titles and abstracts of retrieved records were first screened for inclusion in a full text review. The full texts of potentially relevant studies were then examined to confirm inclusion based on eligibility criteria. Additionally, the references of included studies were also examined to assess the sensitivity of the search strategy and identify any studies that may have been missed. No further studies were identified using this method. Studies published before March 2016 that were chosen for inclusion during the second literature search (conducted by UP) were consistent with those identified during the first literature search (conducted by EP). Results of this second search are presented in the online Supplementary Methods, and in Fig. 1.

Data on study design, sampling framework, inclusion and exclusion criteria, sample characteristics, exposure and outcome definition and ascertainment, covariates, statistical methodology and main results were extracted from included studies. Results of studies were grouped and summarised in terms of overall associations between antibiotic exposure and measures of child body mass, and associations by sex, window of antibiotic exposure, repeated antibiotic exposure, antibiotic class and maternal BMI. Qualitative review and evidence synthesis included an examination of the presence of associations, and their magnitude and statistical strength. The consistency in trends of associations across strata was also considered when examining windows of exposure and repeated exposure to antibiotics. We assessed whether studies adjusted for a number of confounders that may plausibly influence associations between antibiotic use and obesity among children (Box 1; online Supplementary Table S1). Other extracted data on key study characteristics were also assessed in order to understand individual studies’ strengths and limitations. Given the wide variation in study characteristics, we did not perform a meta-analysis of study results.

Box 1. Potential confounders of the association between early life antibiotic use and child obesity

Early childhood infections

Delivery mode

Exclusive breastfeeding

Preterm birth and birth weight

Maternal smoking

Socio-economic status

Maternal BMI

Current wheeze/asthma

Lifestyle (diet and physical activity)

Siblingship

See Supplementary Table S1 for hypothesised pathways.

## Results

### Study characteristics

We identified 13 observational studies, based on a total of 6 81 332 individuals, which permitted assessment of antibiotic exposure before 3 years of age and subsequent measures of body mass or risk of overweight or obesity in childhood (Fig. 1, Table 1) [21–33]. None of the examined studies included assessment of participants’ intestinal microbiota. Study designs included both prospective [22, 26–28, 31, 33] and retrospective [21, 25, 29, 30, 32] cohort studies, and cross-sectional [24] and case–control studies [23]. Apart from one study based in India [27] and two multi-site studies [24, 28], all research was based on North American [23, 25, 29, 30, 32] and European [21, 22, 26, 31, 33] populations (Table 1; online Supplementary Tables S2 and S3).

**Table 1.** Summary of studies included in final review

Study	*N*	Exposure definition and age window	Outcome and age of measurement	Anthropometric reference used for outcome definition	Covariates adjusted for
Saari *et al*. [33]	12 062	Any antibiotic at <24 months	Adjusted difference (*β*) in zBMI at ⩾24 months	Finnish growth reference	Maternal smoking after first trimester, parental relationship, mode of delivery, birth weight and length (length for boys only)
			aOR for overweight at ⩾24 months		
Murphy *et al*. [24]	74 946	Any antibiotic at <12 months	Adjusted difference (*β*) in BMI at 5–8 years		Age, sex, BMI measurement type (parental report or objective measurement) (see Statistical methodology column), maternal smoking, breastfeeding, current wheeze, early life paracetamol use, gross national income (tested for interaction only)
Trasande *et al*. [26]	11 532	Any antibiotic at ⩽24 months	Adjusted difference (*β*) in zBMI at 38 months and 7 years	British 1990 growth reference	Birth weight, maternal parity, maternal race, maternal social class, maternal education, parental BMI (pre-pregnancy and later), first trimester smoking, breastfeeding, timing of introduction of complementary foods, time per day watching television, in car on weekdays and weekends, dietary patterns at 38 months, sleep duration at 7 years
			aOR for overweight at 38 months and 7 years		
Ajslev *et al*. [22]	28 354	Any antibiotic at <6 months^a^	aOR for overweight at 7 years	International Obesity Task Force reference (2007, 2012)	Maternal age, socioeconomic status, pre-pregnancy BMI, gestational weight gain, smoking, paternal BMI, parity, birth weight, sex, exclusive breastfeeding and age at 7 year follow-up
Bailey *et al*. [30]	64 580	Any antibiotic at ⩽23 months	Adjusted hazard ratio for obesity at 24–59 months	Centers for Disease Control and Prevention 2000 reference	Race/ethnicity, age at first primary care visit and number of primary care visits, sex, practice location at first visit, insurance coverage, common childhood infection diagnoses in first 2 years, calendar year and practice of first entry to primary care system, oral steroids and anti-reflux medication
Azad *et al*. [23]	616	Any antibiotic at <12 months	aOR for overweight at 9 years	Centers for Disease Control and Prevention 2000 reference	Sex, birth weight, breastfeeding, maternal overweight, smoke exposure at birth, family income, sibship, diet, physical activity, current asthma, maternal asthma
			aOR for overweight at 12 years		
Rogawski *et al*. [27]	497	Any antibiotic at <6 months	Adjusted difference (*β*) in weight-for-length *z*-score at 6 months to 3 years	World Health Organization 2006 standards	Sex, prior growth *z*-score (measured at the beginning of the month), socioeconomic status, maternal education, household hygiene, household crowding, low birth weight, preterm birth, caesarean delivery, exclusive breastfeeding, number of days with infection/severe illness, number of days with diarrhoea, number of severe diarrhoea events, dehydration during diarrhoea, oral rehydration, hospitalisation events, days with diarrhoea in the previous month
Schwartz *et al*. [25]	1 63 820	Any (and cumulative) antibiotic at ⩽18 years	Adjusted difference (*β*) in BMI at ⩽18 years		Age, sex, race/ethnicity, medical assistance
Gerber *et al*. [29]	38 522	Any antibiotic at <6 months	Adjusted difference in rate of weight gain (%) between 2 and 5 years		Singleton analysis: adjustments made for both mean-level covariates (sex, birth weight, race, Medicaid insurance status, number of siblings, birth year, baseline length, primary care site) and rate-level covariates (as above, excluding primary care site) Twin analysis: sex, birth weight, baseline length
Li *et al*. [32]	2 60 556	Any antibiotic at <12 months among children with infection	aOR for obesity at 2–18 years	Centers for Disease Control and Prevention 2000 reference	Infection type and number of episodes (diagnoses >2 weeks apart), maternal age and race/ethnicity, pre-pregnancy BMI, preterm delivery, sex, low birth weight, maternal antibiotic use and infections during pregnancy, maternal education, marital status, smoking during pregnancy, mode of delivery, timing of initial prenatal care, gestational or pre-existing diabetes, breastfeeding
Scott *et al*. [21]	21 714	Any antibiotic at <24 months	aOR for obesity at 4 years	British 1990 growth reference^b^	Maternal obesity, maternal diabetes, mode of delivery, socioeconomic status, year and country of birth, urban dwelling and sibling obesity
Mbakwa *et al*. [31]	979	Any antibiotic at ⩽10 years	Adjusted difference (*β*) in zBMI at ⩽10 years	Dutch growth reference	Study recruitment group, household size, maternal education, maternal pre-pregnancy weight, maternal pregnancy weight gain, smoking during pregnancy, gestational diabetes, gestational hypertension, mode and place of delivery, sex, birth weight, gestational age, duration of breastfeeding, child's dietary intake and physical activity
			aOR for overweight at ⩽10 years		
Rogawski *et al*. [28]	1954	7-day increase in antibiotic exposure duration at <6 months	Adjusted difference (*β*) in WAZ at 6 months to 2 years	World Health Organization 2006 standards	Site, child's sex, enrolment weight-for-age, WAMI (water, assets maternal education and income) score, household crowding, maternal height, maternal education, characteristics of child's first 6 months (percent days exclusively breast-fed, number of diarrhoea episodes, days with fever or vomiting or respiratory illnesses, presence of acute lower respiratory infection or bloody stools and hospitalisation)

zBMI, body mass index-for-age *z*-score; WAZ, weight-for-age *z*-score; aOR, adjusted odds ratio.

*p < 0.05.

See Supplementary Tables S2 and S3 for full details on characteristics of studies.

Blank cells for anthropometric reference column indicate that none was used.

a Authors reported that questionnaires were only able to capture antibiotics prescribed for ear and lung infection.

b British 1990 growth reference used as part of the UK WHO Term Growth Reference.

Studies varied considerably with respect to a range of methodological aspects, including exposure and outcome definition and ascertainment, analytical strategy and inclusion of covariates (Table 1, online Supplementary Tables S2 and S3). While all studies examined any antibiotic exposure in early life, one study was able to capture only antibiotics prescribed for ear and lung infections [22]. Overall exposure windows of studies ranged from up to 6 months of age [22, 27–29] to up to ⩾3 years of age [25, 31]. Methods of antibiotic exposure ascertainment included parental recall [22, 24, 26–28], prescription data [21, 23, 25, 28–31, 33] and pharmacy data [32]. Outcomes reported ranged from continuous measures including BMI [24, 25] or BMI-for-age *z*-score [26, 31, 33], weight-for-length *z*-score [27] or weight-for-age *z*-score [28] and rate of weight gain [29] to binary measures of overweight [22, 23, 26, 31, 33] or obesity [21, 30, 32]. Outcomes of relative body mass, including BMI-for-age, weight-for-length, weight-for-age and binary classifications of overweight and obesity based on these measures, were expressed using a number of different references (Table 1). Outcome measures were by parental report in three studies [22, 24, 31], and measured in clinical or research settings in the rest [21, 23, 25–30, 32, 33]. Studies employed a variety of analytical methods to assess associations, including those based upon covariance analysis [33], linear regression [24–27, 29], logistic regression [21–23, 26, 32, 33], Cox proportional hazards models [30], generalised estimating equations [28, 31] and longitudinal rate regression [29] (Table 1, online Supplementary Tables S2 and S3). None of the studies adjusted for all variables identified as potentially important confounders (Box 1, online Supplementary Table S3, Table 1).

### Overall associations

Of the 13 studies identified, six reported overall measures of association. Of these, only one reported notable associations, between antibiotic use before 12 months of age and overweight risk at 9 and 12 years of age [adjusted odds ratio (aOR) for overweight at 12 years: 2.56, 95% confidence interval (95% CI) 1.36–4.79] [23]. Five studies, of which two were based on populations of over 38 000 individuals, reported no overall association between antibiotic use and child body mass (Table 2) [22, 27–29, 32]. While all studies involved unmatched cohorts, the two larger studies additionally included matched longitudinal sub-studies of twins discordant for antibiotic exposure, with both confirming the null associations observed in their main analyses [29, 32].

**Table 2.** Overall associations reported between any antibiotic exposure in early life and measures of child body mass

Study	Exposure definition and age window	Outcome and age of measurement	Overall association estimate (95% CI)
Ajslev *et al*. [22]	Any antibiotic at <6 months	aOR for overweight at 7 years	1.04 (0.79–1.37)
Azad *et al*. [23]	Any antibiotic at <12 months	aOR for overweight at 9 years	1.74 (1.04–2.94)*
		aOR for overweight at 12 years	2.56 (1.36–4.79)*
Rogawski *et al*. [27]	Any antibiotic at <6 months	Adjusted difference (*β*) in weight-for- length *z*-score at 6 months to 3 years	−0.10 (−0.23 to 0.02)
Gerber *et al*. [29]	Any antibiotic at <6 months	Adjusted difference in rate of weight gain (%) between 2 and 5 years	0.7 (−0.1 to 1.5)
Li *et al*. [32]	Any antibiotic at <12 months among children with infection	aOR for obesity at 2–18 years	1.01 (0.98–1.04)
Rogawski *et al*. [28]	7-day increase in antibiotic exposure duration at <6 months	Adjusted difference (*β*) in WAZ at 6 months to 2 years	0.03 (0.00–0.05)

zBMI, body mass index-for-age *z*-score; WAZ, weight-for-age *z*-score; aOR, adjusted odds ratio.

*p < 0.05.

See Table 1 and online Supplementary Table S3 for adjustments relating to analyses in each study.

Measures of association are relative to unexposed group unless stated otherwise. Blank cells indicate that no overall association estimate was reported.

### Modification of associations by sex

Sex-specific differences in susceptibility to infections and in drug pharmacokinetics have been previously observed in humans [40–42]. Potential differential effects of antibiotic use on child overweight and obesity risk would thus be important to clarify. Three of four studies stratifying analyses by sex reported associations persisting only in boys following adjustment for covariates such as maternal BMI, smoking or socioeconomic status [23, 24, 33] (Table 3).

**Table 3.** Sex-specific associations reported between any antibiotic exposure in early life and measures of child body mass

Study	Exposure definition	Outcome measure	Association estimate (95% CI or *p* value)
Saari *et al*.[33]	Any antibiotic at <24 months	Adjusted difference (*β*) in zBMI at ⩾24 months	Boys: 0.13 (0.07–0.19)* Girls: 0.07 (0.01–0.13)*
Murphy *et al*. [24]	Any antibiotic at <12 months	Adjusted difference (*β*) in BMI at 5–8 years	Boys: 0.107 (*p* < 0.001)^a^* Girls: −0.008 (*p* = 0.75)^a^^b^
Azad *et al*. [23]	Any antibiotic at <12 months	aOR for overweight at 9 years	Boys: 2.19 (1.06–4.54)*
			Girls: 1.20 (0.53–2.70)
		aOR for overweight at 12 years	Boys: 5.35 (1.94–14.72)*
			Girls: 1.13 (0.46–2.81)^c^
Rogawski *et al*. [27]	Any antibiotic at <6 months	Adjusted difference (*β*) in weight-for-length *z*-score at 6 months to 3 years	Boys: −0.15 (−0.32 to 0.02) Girls: −0.06 (−0.22 to 0.10)

Measures of association are relative to unexposed group unless stated otherwise.

zBMI, body mass index-for-age *z*-score; aOR, adjusted odds ratio.

*p < 0.05.

See Table 1 and online Supplementary Table S3 for adjustments relating to analyses in each study.

a Two-tailed *p* values reported, as 95% CIs were not provided.

b *p* for interaction = 0.002.

c *p* for interaction = 0.05.

### Window of antibiotic exposure

**Fig. 2.**
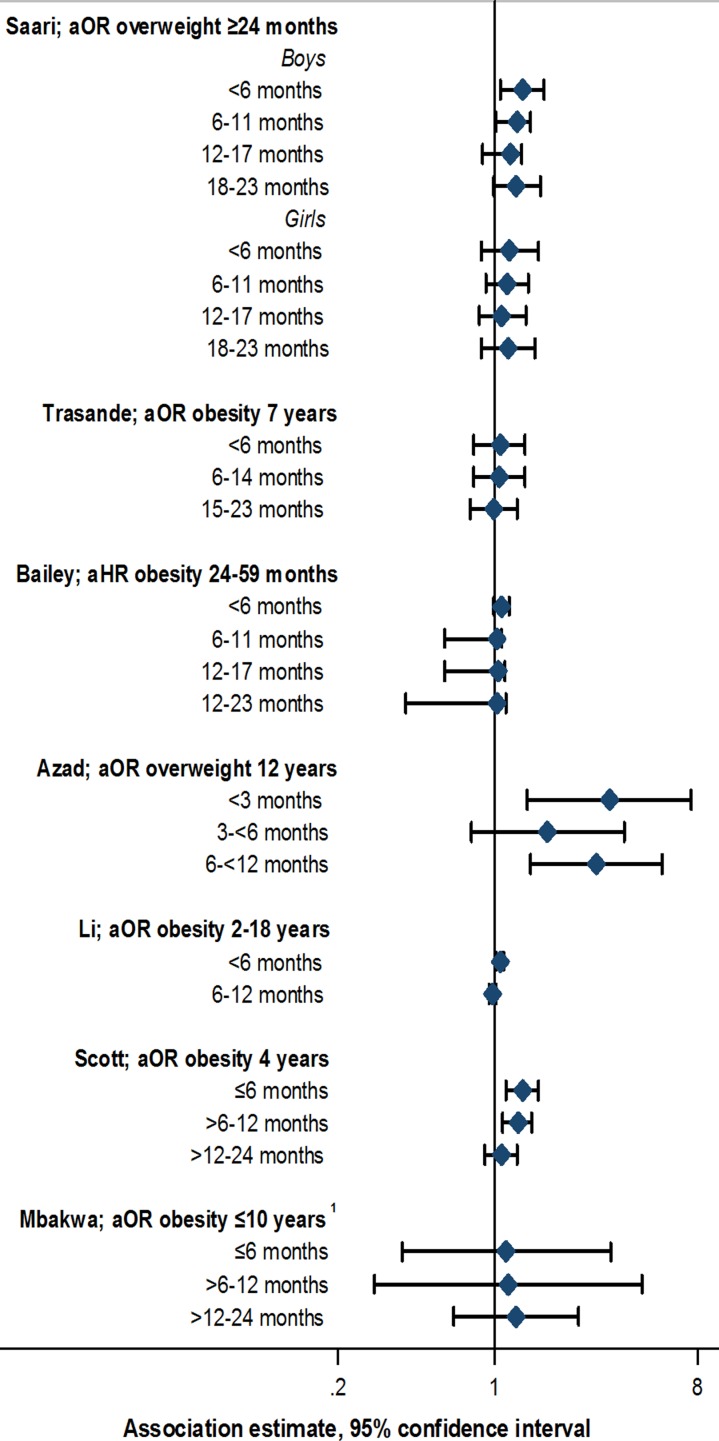
Association between antibiotic exposure in specific age windows and measures of child body mass: selected risk measures reported in reviewed studies. aOR, adjusted odds ratio; aHR, adjusted hazard ratio. ^1^Estimates displayed for children exposed to two or more courses of antibiotics. See online Supplementary Table S3 for adjustments relating to analyses in each study, and online Supplementary Table S4 for additional estimates reported from other studies.

Data from animal models suggest that earlier antibiotic exposure may have the capacity to more notably alter the intestinal microbiome, inducing metabolic changes leading to increased adiposity [43]. Similar effects would be important to clarify in humans. Evidence supporting specific early life windows of antibiotic exposure among studies included in this review was limited. Studies defined windows of exposure differently, often up to 6, 12 or 24 months of age [21–33]. Eight out of 13 studies stratified by exposure window; of these, five reported some evidence of stronger associations with antibiotic exposure in the first 3 to 6 months compared with later periods [21, 23, 26, 32, 33] (Fig. 2, online Supplementary Table S4). However, this pattern was not always consistently observed across multiple outcome measures or time points within the same study [23, 26], and few studies reported notable or consistent trends in associations across consecutive exposure windows [21, 23, 33]. No notable trends in association, or associations with exposure at <6 months of age was noted in two studies [30, 31], whereas another study reported associations between antibiotic use up to 24 months of age, but not up to 6 months of age, and rate of weight gain [29] (online Supplementary Table S4). Statistical tests comparing differences between exposure window categories, or comparing trends across categories, were not performed in any study [21, 23, 26, 29–33].

### Repeated antibiotic exposure

**Fig. 3.**
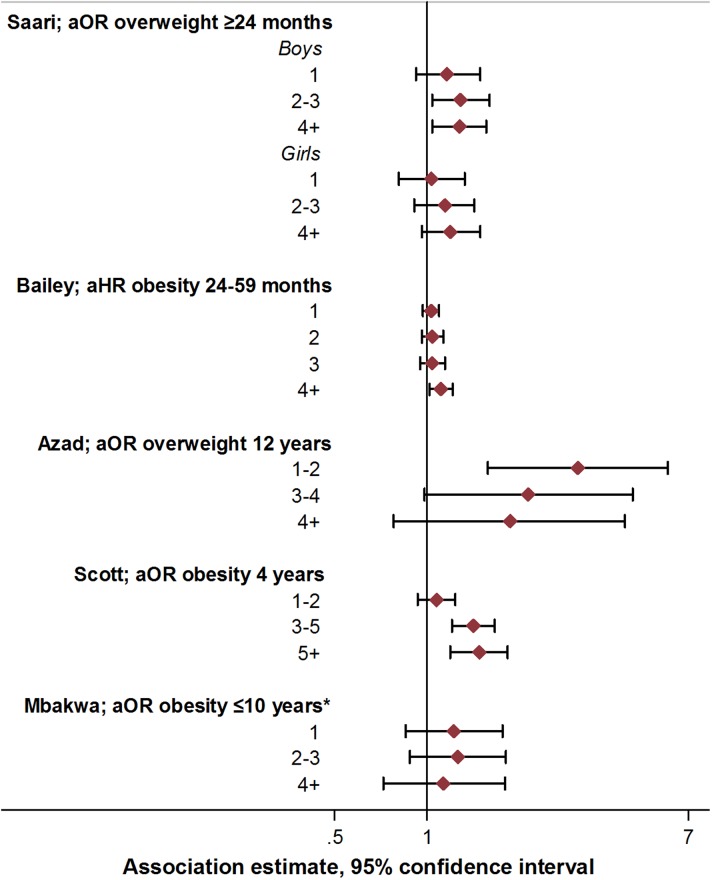
Association between antibiotic course number and measures of child body mass: selected risk measures reported in reviewed studies. aOR, adjusted odds ratio; aHR, adjusted hazard ratio. See online Supplementary Table S3 for adjustments relating to analyses in each study, and online Supplementary Table S5 for additional estimates reported from other studies.

Increased exposure to antibiotics in early life through repeated courses may be expected to result in more pronounced alterations in the intestinal microbiome, with potentially greater metabolic effects. The existence of a dose-dependent relationship between antibiotic use and childhood obesity risk would therefore be important to examine. Seven studies assessed the relation between the number of antibiotic courses and measures of child body mass [21, 23, 27, 29–31, 33] (Fig. 3; online Supplementary Table S5). In four studies, there was some evidence of association between exposure to two or more antibiotic courses and increased measures of body mass, or increasing magnitude of associations with increasing course number [21, 29, 30, 33]. However, trends were not consistent when examined across subgroups within these studies [21, 29, 30, 33]. In two studies, no associations were observed across any category of number of antibiotic courses and measures of body mass [27, 31], while one study reported decreased odds of overweight with exposure to an increasing number of antibiotic courses [23]. Statistical tests for differences or trends across categories of exposure were reported only in this study, and were not notable (*p* for linear trend >0.1 in all cases; Figure 2, online Supplementary Table S5) [23].

### Antibiotic class

Broad spectrum antibiotics are thought to affect a wider range of intestinal microbiota, and thereby more notably alter intestinal microbial composition, with potential subsequent implications for metabolic dysregulation [44]. Evidence on effect modification of obesity risk by antibiotic class in the studies identified here was equivocal (Table 4). Of seven studies stratifying by antibiotic class, three reported stronger associations between broad *v*. narrow spectrum antibiotic use and later child body mass [28, 30, 33], whilst three suggested no differences in strength of associations [29, 31, 32]. One study stratified by anti-anaerobic activity, suggesting increased obesity risk with greater than five courses of antibiotics with anti-anaerobic activity (aOR 1.46, 95% CI 1.09–1.96) [21].

**Table 4.** Association between antibiotic class and measures of child body mass

Study group	Summary measure and outcome	Antibiotic class	Association estimate (95% CI)			
Studies stratifying by exposure window (months)						
			<6 months	6–11 months	12–17 months	18–23 months
Saari *et al*. [33]	aOR for overweight at ⩾24 months	Penicillins – boys	1.28 (0.99–1.62)	1.29 (1.04–1.49)*	1.28 (0.95–1.42)	0.95 (0.78–1.28)
		Cephalosporins – boys	1.44 (0.97–2.35)	1.44 (1.04–1.84)*	1.46 (1.06–1.82)*	1.10 (0.78–1.43)
		Macrolides – boys	1.65 (1.09–2.31)*	1.41 (1.09–1.70)*	1.10 (0.84–1.34)	1.42 (1.18–1.93)*
		Penicillins – girls	1.20 (0.87–1.66)	1.20 (0.97–1.50)	1.04 (0.81–1.33)	1.15 (0.87–1.52)
		Cephalosporins – girls	1.25 (0.67–2.35)	1.18 (0.79–1.76)	1.27 (0.88–1.82)	1.11 (0.75–1.64)
		Macrolides – girls	1.53 (0.90–2.60)	1.11 (0.82–1.50)	1.28 (0.95–1.71)	1.21 (0.88–1.65)
			<6 months	6–11 months	12–17 months	18–23 months
Bailey^a^ *et al*. [30]	Adjusted hazard ratio for obesity at 24–59 months	Broad spectrum^b^ Narrow spectrum^b^	1.12 (1.03–1.19)* 1.03 (0.6–1.08)	1.1 (1.05–1.15)* 0.8 (0.3–1.04)	1.06 (0.99–1.12) 1.01 (0.6–1.06)	1.05 (0.7–1.15) 1.02 (0.4–1.11)
			<6 months	<24 months		
Gerber *et al*. [29]	Adjusted difference in rate of weight gain (%) between 2 and 5 years	Broad spectrum (nonmacrolide) Broad spectrum (macrolide) Narrow spectrum	−0.3 (−2.1 to 1.5) 2.2 (−1.2 to 5.7) 0.4 (−0.6 to 1.3)	2.2 (0.7–3.8)* 2.4 (0.6–4.2)* 1.9 (0.5–3.2)*		
			⩽12 months			
Li *et al*. [32]	aOR for obesity at 2–18 years	Broad spectrum Narrow spectrum	1.02 (0.99–1.05) 1.01 (0.97–1.04)			
Studies stratifying by number of antibiotic courses						
			1–2 courses	3–5 courses	5+ courses	
Scott *et al*. [21]	aOR for obesity at 4 years	Anaerobic coverage^c^ No anaerobic coverage^c^	1.09 (0.95–1.25) 1.09 (0.93–1.27)	1.45 (0.91–1.68) 1.24 (0.91–1.68)	1.46 (1.09–1.96)* 1.00 (0.63–1.60)	
			1 course	2+ courses		
Mbakwa *et al*. [31]	Adjusted difference (*β*) in zBMI at ⩽10 years aOR for overweight at ⩽10 years	Broad spectrum *β*-lactams Narrow spectrum *β*-lactams Macrolide Antimetabolite Others Broad spectrum *β*-lactams Narrow spectrum *β*-lactams Macrolide Antimetabolite Others	−0.05 (−0.16 to 0.06) 0.08 (−0.10 to 0.26) 0.09 (−0.05 to 0.23) −0.05 (−0.30 to 0.19) 0.13 (−0.29 to 0.56) 0.95 (0.68–1.34) 1.00 (0.57–1.76) 1.29 (0.89–1.86) 0.83 (0.33–2.11) 1.62 (0.52–5.06)	0.07 (−0.04 to 0.19) 0.16 (−0.18 to 0.49) −0.07 (−0.28 to 0.14) −0.02 (−0.41 to 0.37) −0.16 (−0.51 to 0.19) 1.20 (0.86–1.67) 0.41 (0.06–2.63) 0.64 (0.35–1.16) 0.67 (0.19–2.31) 0.50 (0.21–1.20)		
			1 or 1+ courses	2+ courses		
Rogawski *et al*. [28]	Adjusted difference (*β*) in WAZ at 6 months to 2 years	Metronidazole Macrolides Cephalosporins Penicillins Fluoroquinolones Sulphonamides	0.14 (−0.01 to 0.29) 0.09 (−0.03 to 0.21) 0.03 (−0.09 to 0.16) 0.09 (−0.02 to 0.19) 0.08 (−0.14 to 0.30) 0.03 (−0.09 to 0.15)	0.24 (0.04–0.43)* 0.23 (0.05–0.42)* 0.19 (0.04–0.35)* 0.07 (−0.04 to 0.18)		

zBMI, body mass index-for-age *z*-score; WAZ, weight-for-age *z*-score; aOR, adjusted odds ratio.

Measures of association are relative to the unexposed group, and are presented with 95% CIs unless stated otherwise.

*p < 0.05.

See online Supplementary Table S3 for adjustments relating to analyses in each study.

a Estimates read off graph.

b Penicillin and amoxicillin classified as narrow spectrum operationally, all other agents considered broad.

c Antibiotics with anaerobic coverage: penicillins, imidazoles, lincosamides, tetracyclines; without anaerobic coverage: cephalosporins, macrolides, sulpha-containing agents, isoniazid, rifampicin, fluoroquinolones, aminoglycosides.

### The effect of maternal BMI

Intestinal microbial composition in pregnant women has been shown to differ by weight status, and to influence the offspring intestinal microbiome [45]. Such resulting variations are hypothesised to lead to differential weight-related responses to antibiotic exposure among children of overweight *v*. normal weight mothers [45]. Two studies examined maternal weight as a potential effect modifier [22, 26]. One study reported associations between antibiotic use before 6 months of age and overweight at 7 years of age only among children of normal weight mothers (aOR 1.54, 95% CI 1.09–2.17) [22]. The other study observed similar associations with child overweight at 38 months of age; however, these did not persist to overweight at 7 years [26].

### Addressing confounding by clinical indication

Confounding by clinical indication occurs when the association between a clinical treatment and outcome is explained by the disease for which treatment was indicated [46]. Measures to identify and address confounding by indication are thus crucial in studies examining clinical treatments [46]. Four studies here considered infection status, with three stratifying by, or adjusting for, infection type or severity [27, 28, 30]. Only one study clearly examined the influence of the indication for antibiotic use on child obesity risk, reporting associations between the presence of early life infection and obesity (aOR 1.25, 95% CI 1.20–1.29), but not between antibiotic use and obesity among children with infection (aOR 1.01, 95% CI 0.98–1.04) [32]. Notably, including both healthy children and those with untreated infection in the reference group resulted in associations between antibiotic use and child obesity that were comparable with those observed in other studies (aOR 1.20, 95% CI 1.17–1.22) [32], suggesting that inappropriate accounting for confounding by indication may be an important limitation of other studies [21–26, 29, 31, 33] (online Supplementary Tables S4 and S5).

### Quantification of weight gain

Four studies examining measures of relative BMI or weight as outcomes (Table 1) quantified the absolute magnitude of weight gain associated with antibiotic exposure [25–27, 29]. Estimates ranged from increases of 0.001 and 0.032 kg at 2 years of age among boys and girls exposed to antibiotics in the first 6 months of life [27] to a maximum weight gain of 1.50 kg by 15 years of age associated with exposure to macrolides [25]. One study estimated 0.09 kg increased weight among 38 month old children exposed to antibiotics up to 2 years of age [26], whilst another estimated a 0.15 kg increase between 2 and 5 years of age attributable to antibiotic use in the first 6 months of life [29]. In all cases, reported gains in weight approximated to <2.9% of the age-specific population median, proportions that would be unlikely to notably alter risk of adverse outcomes (online Supplementary Table S6).

## Discussion

Clarifying the existence and nature of associations between antibiotic use in early life and subsequent risk of child obesity is essential to guide appropriate policies and strategies relating to child health and to use of antibiotic medications. We examined observational studies exploring the relation between early childhood antibiotic use and measures of child body mass. The totality of evidence was inconsistent, with key differences in study design potentially contributing to varying conclusions. Overall, there was no strong evidence supporting clinically relevant associations between antibiotic exposure and body mass in childhood. Further comprehensive and comparable studies using routine clinical data may help clarify this association.

Although the exact measures varied by study, associations reported between antibiotic use and child body mass were generally not appreciable in terms of effect size and statistical strength. In certain studies, associations were only apparent when examining exposure to four or more antibiotic courses [21, 30]. Trends or patterns, such as those relating to consecutive exposure windows or antibiotic class, were often inconsistent across studies or study subgroups [21, 23, 28–33]. Notably, studies quantifying observed associations did not indicate important absolute gains in weight related to antibiotic exposure, suggesting that if this association does exist, it is not likely to be clinically relevant [25–27, 29].

Findings reported in studies must also be considered within the context of their specific limitations. These limitations ranged from small population or subgroup sample sizes [23, 27, 31] to errors in ascertainment of antibiotic exposure measured through parental recall [22, 24, 26, 27] or by prescriptions [23, 25, 27, 29, 30] or examination of only specific kinds of antibiotics [22], and the inability to measure adherence to treatment. Such errors in ascertainment of antibiotic exposure may limit the ability of studies to detect associations. Not all studies controlled for a comprehensive range of relevant confounders, and most importantly, most studies did not sufficiently examine confounding by clinical indication [21–31, 33, 46]. We found only one study to have accounted for this, which reported no association between antibiotic use and obesity risk among children with infection, and demonstrated the potential for observing erroneous positive associations when including uninfected children in the reference group [32]. These findings indicate that associations reported in other studies must be cautiously interpreted, and highlight the need for further, more robust evidence [21–31, 33].

In this review, we were unable to synthesise effect estimates meaningfully given the high degree of heterogeneity among studies, which included differences in study populations and settings, observation windows, definitions of exposures and outcomes, inclusion of potential confounders and effect modifiers and choice of analytic approaches. To improve comparability and enable synthesis of data in meta-analyses, future studies should ideally: (i) define all exposures and outcomes, including their respective time windows, consistently with previous studies, (ii) consider using multiple analytic methods as employed in previous research and (iii) control for all confounding factors included in previous studies, including accounting for confounding by indication. As stronger evidence emerges regarding the characterisation and contribution of related measures in childhood, such as waist circumference, to future disease risk, studies may also consider such measures as potentially important outcomes [23, 47]. Given the effect sizes observed here, future studies must be based on large populations in order to allow for more precise estimation of association. Given this required scale and complexity, such analyses would be best facilitated by the use of routine clinical data captured in electronic health records, as demonstrated in key studies reviewed here [21, 23, 25, 29, 30, 32]. Electronic health records provide a platform for obtaining large-scale, longitudinal patient data, as well as facilitating the collection of information on infection type, test results, treatment dose, duration and response and other potential confounding factors. They may additionally be utilised to prospectively recruit patients into future, large scale studies.

The evidence reviewed here is insufficient to justify the risk of child obesity as an important reason to limit antibiotic use. However, multiple other reasons exist for improving monitoring and management of antibiotic use, including the development of antibiotic resistance. The importance of antibiotic stewardship programmes to achieve appropriate use of antibiotics is increasingly being recognised, with recent evidence suggesting notable effects of such initiatives on overall antibiotic use, infection incidence and other patient outcomes in hospitals [48–50]. The expansion of these programmes may help address antibiotic resistance on a wider scale, and thus contribute to reducing associated morbidity and mortality [51]. However, the evidence here does not support a tangible effect of such programmes on the current burden of child obesity, and suggests that resources aimed towards child obesity prevention would be better placed in existing strategies and initiatives targeting appropriate nutrition and physical activity in early life and later childhood [52].

We followed a systematic literature search and selection method to identify all relevant studies investigating the relation between early life antibiotic use and child obesity, and present here a detailed qualitative synthesis of identified studies. However, certain limitations must be considered. We included only English language publications in our search, and are unable to rule out publication bias, although the presence of such bias would support our interpretation of the current evidence. The inability to quantitatively synthesise effect estimates limits our ability to present more definitive conclusions, and points towards the need for more harmonised methods to assess this association. Finally, this review examined only exposure to antibiotics for medical use, and did not capture potential exposure through other methods, such as low-dose antibiotics present in food. Although this has been recognised as a potentially important source of exposure [11, 53], few studies, to our knowledge, have quantified it and explored its association with outcomes in humans, and it remains a potentially important area for future research.

In this review, we observed no robust evidence implicating antibiotic use as an important risk factor for child obesity, with notable heterogeneity among studies limiting more conclusive interpretations. Further more detailed, comparable studies are required to clearly determine the existence of such associations. Regardless, there is a well-justified need to achieve more appropriate use of antibiotics [51]. Furthermore, multiple comprehensively researched risk factors may be targeted to address child obesity [52]. Efforts in these directions will be key for addressing both these concerning public-health issues on a large scale.

## Supplementary material

For supplementary material accompanying this paper visit https://doi.org/10.1017/gheg.2018.16. click here to view supplementary material
