# Supplementary material for: Association between early life antibiotic use and childhood overweight and obesity: a narrative review
Source: Glob Health Epidemiol Genom. 2018 Oct 24;3:e18. doi: 10.1017/gheg.2018.16 (PMC6218928; doi:10.1017/gheg.2018.16)
Supplement: Supplementary file 1 [file S2054420018000167sup001.zip › S2054420018000167sup001/180831_2Child_Abx_OB_SuppMethods.docx]

**Association between early life antibiotic use and childhood overweight and obesity: a narrative review**

**Supplementary Methods**

Literature searches were performed using PubMed, Web of Science and the Cochrane Library to identify studies published before October 2017 that assessed the relation between early childhood antibiotic use and measures of child body mass. A summary of the search terms used and records identified and examined is presented below.

**Literature search terms**

PubMed

(BMI[title/abstract] OR "body mass index"[MeSH Terms] OR "obesity"[MeSH Terms] OR "overweight"[MeSH Terms] OR ("weights and measures"[MeSH Terms] OR "body weight"[MeSH Terms]) OR "anthropometry"[MeSH Terms] OR "growth"[MeSH Terms] OR "body weight"[MeSH Terms] OR "body size"[MeSH Terms] OR growth curve[title/abstract] OR "growth charts"[MeSH Terms] OR "weight gain"[MeSH Terms] OR (("human body"[MeSH Terms] OR ("human"[All Fields] AND "body"[All Fields]) OR "human body"[All Fields] OR "body"[All Fields]) AND "molecular weight"[MeSH Terms]) OR "adiposity"[MeSH Terms]) AND ("anti-bacterial agents"[MeSH Terms] OR "anti-bacterial agents"[MeSH Terms] OR anti-bacterial[title/abstract]) AND (childhood[title/abstract] OR "infant"[MeSH Terms] OR postnatal[title/abstract] OR "adolescent"[MeSH Terms] OR early-life[title/abstract] OR early[title/abstract] OR "child"[MeSH Terms])

Filter – only English

Web of Science

#1: (TS= (BMI) OR TS= (body mass index) OR TS= (obesity) OR TS= (overweight) OR TS= (weight) OR TS= (anthropometry) OR TS= (growth) OR TS= (body weight) OR TS= (body size) OR TS= (growth curve) OR TS= (weight gain) OR TS= (body mass) OR TS= (adiposity)) AND LANGUAGE: (English) AND DOCUMENT TYPES: (Article)

Indexes=SCI-EXPANDED, SSCI, A&HCI, CPCI-S, ESCI Timespan=All years

#2: (TS= (early antibiotic*) OR TS= (childhood antibiotic*) OR TS= (infant antibiotic*) OR TS= (postnatal antibiotic*) OR TS= (early-life antibiotic*) OR TS= (early antibacterial) OR TS= (early anti-bacterial) OR TS= (childhood antibacterial) OR TS= (childhood anti-bacterial) OR TS= (infant antibacterial) OR TS= (infant anti-bacterial) OR TS= (early-life antibacterial) OR TS= (early-life anti-bacterial)) AND LANGUAGE: (English) AND DOCUMENT TYPES: (Article)

Indexes=SCI-EXPANDED, SSCI, A&HCI, CPCI-S, ESCI Timespan=All years

#2 AND #1

Cochrane Library

#1 MeSH descriptor: [Body Mass Index] explode all trees

#2 MeSH descriptor: [Overweight] explode all trees

#3 MeSH descriptor: [Obesity] explode all trees

#4 MeSH descriptor: [Weight Gain] explode all trees

#5MeSH descriptor: [Body Weight] explode all trees

#6 MeSH descriptor: [Anthropometry] explode all trees

#7 MeSH descriptor: [Body Weight Changes] explode all trees

#8 MeSH descriptor: [Growth] explode all trees

#9 MeSH descriptor: [Growth Charts] explode all trees

#10 MeSH descriptor: [Adiposity] explode all trees

#11 MeSH descriptor: [Anti-Bacterial Agents] explode all trees

#12 MeSH descriptor: [Child] explode all trees

#13 MeSH descriptor: [Infant] explode all trees

#14 (#1 or #2 or #3 or #4 or #5 or #6 or #7 or #8 or #9 or #10) and (#11) and (#12 or #13)
