# Supplementary material for: Association between early life antibiotic use and childhood overweight and obesity: a narrative review
Source: Glob Health Epidemiol Genom. 2018 Oct 24;3:e18. doi: 10.1017/gheg.2018.16 (PMC6218928; doi:10.1017/gheg.2018.16)
Supplement: Supplementary file 1 [file S2054420018000167sup001.zip › S2054420018000167sup001/180831_3Child_Abx_OB_SuppTables.docx]

**Association between early life antibiotic use and childhood overweight and obesity: a narrative review**

**Supplementary Tables**

| **Supplementary Table S1. Potential confounders of the association between early life antibiotic use and child obesity and hypothesized underlying pathways.** | |
| --- | --- |
|  |  |
| **Early childhood infections** | Early childhood infections, which antibiotics are indicated for, can result in perturbed nutrient absorption of enteric as well as systemic inflammation, which is associated with growth (1). Longitudinal studies have also reported a residual effect of poor childhood growth and childhood illness on metabolic syndrome characteristics (2, 3). |
|  |  |
| **Delivery mode** | Children born by Caesarean section have increased risk of obesity in later childhood, relative to those born by vaginal birth (4). Caesarean delivery has also been associated with asthma, which results in respiratory symptoms for which antibiotics might be indicated. Asthma has also been associated with overweight (5, 6). |
|  |  |
| **Exclusive breastfeeding** | Exclusive breastfeeding has known protective effects against overweight as well as childhood infections that antibiotics are indicated for (7). |
|  |  |
| **Preterm birth and birth weight** | The aberrant, rapid “catch-up” growth patterns associated with preterm birth and low birth weight are thought to result in metabolic adaptions that increases risk for later overweight (8). Evidence suggests an association between low birth-weight and reduced diversity in microbiota composition (9). Both factors are thought to make children more susceptible to infections, which is associated with antibiotic exposure and altered growth (9). |
|  |  |
| **Maternal smoking** | Prenatal and postnatal passive exposure to smoking is associated with increased incidence of asthma and respiratory symptoms such as wheezing in children, which antibiotic might be indicated for (10). Maternal smoking is also associated with childhood overweight (11). |
|  |  |
| **Socio-economic status (SES)** | SES is associated with childhood overweight, through complex demographic and life-style factors, including diet (12). SES may also be associated with antibiotics through parental receptivity to medicine (13, 14). |
|  |  |
| **Maternal BMI** | Maternal overweight is associated with child overweight through both behavioural and demographic pathways (15), as well as biological mechanism such as vertical transmission of an obesogenic microbiome (16, 17). Maternal underweight is associated with preterm birth and low birth weight (see above) (18). |
|  |  |
| **Current wheeze/ asthma** | Asthma results in respiratory symptoms, including a wheeze that antibiotics are often indicated for. Asthma has been associated with childhood overweight (19). |
|  |  |
| **Lifestyle** | Lifestyle factors such as diet and physical activity have well-studied contributions to childhood overweight (20), and are thought to influence immunity to infection (21). |
|  |  |
| **Siblingship** | Children with siblings have a higher exposure to infections, that antibiotics are indicated for, and are also at an increased risk for overweight (22). |
| BMI: Body mass index | |

| **Supplementary Table S2. Summary of studies included in final review – description of study population and design.** | | | | | | | | |
| --- | --- | --- | --- | --- | --- | --- | --- | --- |
| **Article** | **Location** | **Sample** | **Sample size** | **Follow-up, % completed** | **Exposure** | **Exposure ascertainment** | **Outcome** | **Outcome ascertainment** |
| Saari et al. 2015 (23) | Espoo, Finland | Population-based longitudinal birth cohort attending primary  care facilities as part of the Finnish growth reference study population | 12,062 | Start:  2003-2007  Follow-up: till ≥24 months Completed: 81.7% | Antibiotic exposure in the first 24 months of life, by antibiotic class, exposure window, number of courses | Antibiotic prescription data retrieved from Social Insurance Institution of Finland database. Extracted prescription information for children from birth to 24 months of age | - BMI at ≥24 months of age  - Risk of overweight and obesity at ≥24 months of age | Anthropometric data obtained from electronic health records for primary care visits after 24 months. BMI, length/height measures and birth size data transformed to z-scores for age according to Finnish growth references. Overweight and obesity defined by national cut-offs for zBMI based on  age-specific percentile curves passing through adult values for 25 kg/m^2^ and 30 kg/m^2^ respectively |
| Murphy et al.  2014 (24) | 31 centres in 18 countries | Multi-centre, multi-country, multi-phase cross-sectional  study (The International Study of Asthma and Allergies in Childhood Phase three). Children aged 5-8 years selected from random sample of schools in defined geographical region | 74,946 | Undertaken: 2000-2003  Completed: 82.03% | Antibiotic use in first 12 months of life as binary variable | Standardised core questionnaire included questions on the use of any antibiotics the first 12 months of life, which was completed by parents | BMI at 5-8 years of age | Parents reported child’s current anthropometric data on questionnaires used to calculate BMI.  Some centres measured height and weight objectively, but without standardisation. |
| Trasande et al. 2012 (25) | Avon, UK | Population-based longitudinal birth cohort, Avon Longitudinal Study of Parents and Children, of children born in Avon, UK 1991-1992 | 11,532 | Start: 1991-1992  Follow-up: 7 years  Completed: 79.31% | Antibiotic exposure in the first 24 months of life as binary variable | Three postal questionnaire used to assess exposures: at 6 months (for <6 month interval), at 15 months (for 6-14 months interval) and at 24 months (for 15-23 months intervals) | - Weight-for-length z score at 6-8 weeks, 38-44 weeks, 77-106 weeks.  - BMI z score at 38 months and 7 years  - Risk of overweight at 38 months and 7-years | Anthropometric data for time points 6 weeks, 10 months, 20 months and 38 months extracted from health visitor records. Measurements at seven-years assessed by cohort researcher in clinic visit. WHO weight-for-length Z-score used until 2- years of age. Thereafter, 1990 UK BMI Z-score till 7-years. Overweight defined as zBMI in 85-94th percentile for age and sex, and obesity as zBMI>95th percentile for age and sex. |
| Ajslev et al.  2011 (22) | Denmark | Longitudinal prospective study, the Danish National Birth Cohort, which captured 30% of the Danish pregnant population at the time | 28,354 | Start: 1997-2002  Follow-up:  7 years  Completed: n/a (ongoing) | Antibiotic exposure in first 6 months of life as binary variable (antibiotics found to be prescribed either for lung or ear infection) | Information on exposure status obtained from telephonic interview at 6 months | Risk of categorical overweight at 7 years of age | Paper or internet questionnaire at 7-year follow-up to collect anthropometric data. BMI classified according to International Obesity Task Force's age- and sex-specific BMI cut-off scores for overweight, utilizing averaged centile curves that pass through the adult values for 25 and 30 kg/m^2^ respectively for overweight and obese |
| Bailey et al. 2014 (26) | Urban Philadelphia, Pennsylvania, New Jersey, Delaware, USA | Cohort study with electronic health records in the primary care network affiliated with the Children's Hospital of Philadelphia including clinics and private practices in. All children with annual visits at 0-11 and 12-23 months as well as one or more visit at 24-59 months enrolled | 64,580 | Start: 2001-2003  Follow-up: maximum 59 months  Completed: 73.53% | Antibiotic exposure in the first 23 months of life. Exposure information collected by class, exposure window and course number | Outpatient prescriptions and patient-reported records; antibacterial use recorded at primary care visits | Risk of obesity at 24-59 months of age | Anthropometric data at the first visit after 24 months retrieved from electronic health records. BMI compared to 2000 National Health and Nutrition Examination Survey’s growth reference. Overweight defined as BMI in 85-94th percentile for age and sex, and obesity as BMI >95th percentile for age and sex. |
| Azad et al. 2014 (27) | Manitoba, Canada | Nested case-control study of the longitudinal birth cohort (Study of Asthma, Genes and the Environment). Surveyed all children 7 years of age in cohort still residing in area for permission to enrol in nested case-control | 616 | Cohort started: 1995  Children surveyed for nested study enrolment: 2002  Follow-up: 5 years;  3598 of 13980 surveys returned, 723 enrolled | Antibiotic exposure during first 12 months of life as binary variable. Exposure information collected by class, exposure window and course number | Prescription records retrieved from provincial health-care database records | Risk of categorical overweight at 9 and 12 years of age | Anthropometric data taken at clinical assessment points at 9 years and 12 years of age. BMI used as a measure of total adiposity at ages 9 and 12. Overweight defined as BMI >85th percentile for age and sex according to US CDC growth charts. |
| Rogawski et al. 2015 (28) | Vellore, India | Community-based birth cohort enrolled to estimate the prenatal and postnatal determinants of illnesses of children residing in a semi-urban informal settlement. Permanent resident pregnant women recruited via door-to-door survey in four regions of semi-urban area around Vellore | 497 | Start: 2009  Follow-up: 2 years  Completed: 91.8% | Antibiotic exposure during first 6 months of life as binary variable | Fieldworkers interviewed caregivers twice/ week  from birth to 3 years. Antibiotic exposure defined at monthly intervals as antibiotic use for any illness. Included caregiver reported antibiotic use for diarrhoea during home visits by fieldworker, or if prescription was recorded in study clinic for all illness. | Short and long term effects on HAZ, WAZ, WHZ | Anthropomorphic data recorded every month at the study clinic, using standardised procedures. WAZ, HAZ and WHZ z-cores calculated using 2006 WHO child growth standard as reference population. |
| Schwartz et al. 2016 (29) | Central and North-eastern Pennsylvania, USA | Population-based open cohort from Geisinger Health System’s electronic health record of children between three and 18 years old with primary-care provider in GH system | 163,820 | Data collected from 2001 to 2012 | Antibiotic exposure in the year before each BMI measure (to define recent exposure) and cumulative number of antibiotic exposures | Prescription orders collected from e-records. Only one order per day was counted. | BMI trajectory growth curves | BMI computed by clinic-based measures. Untransformed BMI trajectories modelled by age (duration from birth to each BMI measurement) |
| Gerber et al. 2016 (30) | Pennsylvania, New Jersey, and Delaware, USA | Retrospective cohort of children attending paediatric primary care practices served by a common electronic health record provider (Epic Systems); including a longitudinal cohort of singleton births, and a matched longitudinal cohort of twins discordant for antibiotic exposure | 38,522 singleto-ns, 96 twins (42 pairs) | Data collected from 2001 to 2012 | Primary: antibiotic exposure during first 6 months of life  Secondary: antibiotic exposure during first 24 months of life | Prescription records for systemic (oral, intramuscular, intravenous) antibacterial agents, of any duration except in the case of oral medication for less than 3 days. | Growth trajectory via weight change, based on longitudinally measured weight in the first 8 years of life | Weight measurements from preventative health encounters (well-child visits) |
| Li et al. 2016 (31) | Northern California, USA | Retrospective birth cohort of children attending health institutions under the Kaiser Permanente Northern California (KNPC) integrated managed care consortium, with KPNC electronic medical records; including a longitudinal cohort of singleton births, and a matched longitudinal cohort of twins discordant for infection and antibiotic exposure | 260, 556 singleto-ns, 1094 twins (547 pairs) | Data collected from Jan 1, 1997 to March 31, 2015 | Infection status in the first 12 months of life  Antibiotics in the first 12 months of life (excluding exclusively topical antibiotics or antiviral/antifungal/antiparasitic drugs, and antibiotic use without infection diagnosis) | KPNC electronic medical records capturing all clinical diagnoses of infections from outpatient visits, hospital admissions, emergency department and urgent care clinic visits, telephone consultations  KPNC pharmacy database records capturing all prescription medication including antibiotics, dates of prescription and dispensing, doses, daily amount, days of supply and route of administration (oral, injection or topical) | Obesity status at 2-18 years of age, defined using Centers for Disease Control and Prevention 2000 growth reference | BMI calculated from height and weight measurements taken during routine clinical visits |
| Scott et al. 2016 (32) | UK | Retrospective cohort study of children in The Health Improvement Network, registered within 3 months of birth and with complete follow up to 4 years of age, with measures recorded in a common electronic medical record (EMR) system (Vision EMR) | 21,714 | Data collected from 1995-2013 | Antibiotic use before 2 years of age | Any prescription for a systemic antibiotic from enrolment to 2 years, as captured in EMR system (number of prescriptions used to define number of antibiotic courses) | Obesity risk at 4 years of age, defined using the UK-World Health Organization term growth reference | BMI calculated from height and weight measures taken within one week of each other |
| Mbakwa et al. 2016 (33) | The Netherlands | Cohort study of children in the KOALA (Child, parents and health: lifestyle and genetic constitution) Birth Cohort Study, whose parents consented to having information on their child’s antibiotic use obtained from their general practitioner (GP) | 979 | Data collected from October 2002-2014 | Antibiotic use up to 10 years of age | Any prescription for antibiotics prescribed in the general practice setting, as provided by the GP | Age- and sex-specific BMI z-scores (and dichotomised to overweight), defined using the Dutch Growth Study population reference | BMI calculated from height and weight measured and reported by parents in postal questionnaires, administered at 7 time points between infancy and childhood |
| Rogawski et al. 2017 (34) | 8 sites in 8 lower and middle income countries | Prospective birth cohort of children recruited into the MAL-ED (Etiology, Risk Factors, and Interactions of Enteric Infections and Malnutrition and the Consequences for Child Health) study, enrolled within 17 days of birth | 1954 | Data collected from November 2009-February 2014 | Oral or injected antibiotics in the first 6 months of life | Caregiver report during fortnightly follow-ups, confirmed by medication packaging and prescriber documentation | Age- and sex-specific WAZ and HAZ at 6-24 months of age, defined using the World Health Organization 2006 standards | Weight measured during monthly home visits (standardised procedures not specified) |
| BMI: body mass index; zBMI: BMI z-score; HAZ: height-for-age z-score; WAZ: weight-for-age z-score; WHZ: weight-for-height z-score. | | | | | | | | |

| **Supplementary Table S3. Further population characteristics and analytic methods used in reviewed studies.** | | | | | | |
| --- | --- | --- | --- | --- | --- | --- |
| **Study** | **Male participants**  **(%)** | **Antibiotic exposure prevalence**  **(%)** | **Overweight and obesity prevalence**  **(%)** | **Measure of association** | **Statistical methodology** | **Covariates included in adjusted models** |
| Saari et al. 2015 (23) | 6,114/12,062  (50.7) | <24 months:  9,236/12,062  (76.6) | Overweight:  2,094/12,062  (17.36) | Difference in adjusted zBMI-for-age  Adjusted OR for overweight | Covariance analysis method with random effects for subjects used to compare zBMI between exposed and unexposed as a binary variable, exposure window and class  Logistic regression used for categorical overweight risk | Maternal smoking after first trimester, parental relationship, mode of delivery, birth weight and length (length for boys only) |
| Murphy et al. 2014 (24) | Not reported | <12 months:  Range: 22% in Taiwan to 76% in Thailand | Not provided | Difference in adjusted BMI | General linear mixed model with sex within centre as random effect and age, sex, BMI measurement type and early antibiotic as fixed effects | Age, sex, BMI measurement type (parental report or objective measurement) (see Statistical methodology column), maternal smoking, breastfeeding, current wheeze, early-life paracetamol use, gross national income (tested for interaction only) |
| Trasande et al. 2012 (25) | Not reported | <23 months:  6,599/8,881 (74.3) | Overweight at 38 months: 1,700/9,443 (18)  Overweight at 7 years: 1,962/8,210 (23.9)  Obesity at 38 months: 793/9,443 (8.4)  Obesity at 7 years: 706/8,210 (8.6) | Difference in adjusted zBMI-for-age and sex  Adjusted OR for overweight and obesity | Mixed models with subjects as random effects separately for three exposure windows – linear models for continuous zBMI and logistic for categorical overweight/obesity, with interaction terms for maternal weight included. Each outcome time-point assessed separately | Birth weight, maternal parity, maternal race, maternal social class, maternal education, parental BMI (pre-pregnancy and later), first trimester smoking, breastfeeding, timing of introduction of complementary foods, time per day watching television, in car on weekdays and weekends, dietary patterns at 38 months, sleep duration at 7 years |
| Ajslev et al. 2011 (22) | Not reported | <6 months:  1,628/22,368 (72.8) | Overweight at 7 years:  2,333/28,354 (8.23)  Obese at 7 years:  378/28,354 (1.33) | Adjusted OR for overweight | Multiple logistic regression for categorical overweight risk, with interaction terms for maternal weight | Maternal age, socioeconomic status, pre-pregnancy BMI, gestational weight gain, smoking, paternal BMI, parity, birth weight, sex, exclusive breastfeeding and age at 7 year follow-up |
| Bailey et al. 2014 (26) | 33,353/65,480  (51.0) | ≤23 months:  45,181/65,480 (69.0) | Overweight or obese at:  2 years: 23%  3 years: 30%  4 years: 33% | Adjusted rate ratio for obesity | Cox proportional hazards model | Race/ethnicity, age at first primary care visit and number of primary care visits, practice location at first visit, insurance coverage, common childhood infection diagnoses in first two years, calendar year and practice of first entry to primary care system, oral steroids and anti-reflux medication, sex |
| Azad et al. 2014 (27) | 346/616  (56.17) | ≤12 months:  438/616 (71.1) | Overweight at age 9:  181/616 (29.4)  Overweight at age 12:  121/431 (28.1)  High central adiposity at age 12: 145/428 (33.9) | Adjusted OR for overweight and high central adiposity | Multiple logistic regression for linear trend for cumulative exposure. Association stratified by sex and maternal overweight, based on previous associations in literature | Sex, birth weight, breastfeeding, maternal overweight, smoke exposure at birth, family income, sibship, diet, physical activity, current asthma, maternal asthma |
| Rogawski et al. 2015 (28) | 263/456 (52.9) | <6 months:  262/456 (57.5) | Wasting, based on weight-for-height data, at 27 months:  68/456 (15) | Adjusted absolute change in z-score | Short-term effects: General linear regression to model WAZ, HAZ and WHZ at monthly intervals for 0-5 months, with generalized estimating equations. Long-term effects: Created growth curves after 6 months of age by grouping measurements of height and weight by month, averaging across children given the same number of antibiotic courses before 6 months of age. Longitudinal linear regression with GEE used to model WAZ, HAZ AND WHZ after 6 months as function of antibiotic use in first 6 months | Sex, prior growth z-score (measured at the beginning of the month), socioeconomic status, maternal education, household hygiene, household crowding, low birth weight, preterm birth, caesarean delivery, exclusive breastfeeding, number of days with infection/severe illness, number of days with diarrhoea, number of severe diarrhoea events, dehydration during diarrhoea, oral rehydration, hospitalization events, days with diarrhoea in the previous month |
| Schwartz et al. 2016 (29) | 96,654/163,820  (50.2) | 59% with at least one order while under care i.e. captured in system | Not reported | Beta coefficients for change in BMI | Models included different fixed-effect terms for age, including age, age^2^, age^3^, sex, race/ethnicity, and Medical Assistance), as well as random intercept terms for age and age^2^ which were allowed to covary with unstructured covariance. Residual variance was allowed to vary by age group. Models included cross-products of sex and Medical Assistance with the three age terms as well as race/ethnicity and age terms as fixed effects.  BMI in a given year regressed on antibiotic history up to that time by three antibiotic main effect terms – one to capture recent exposure i.e. 0/1 indicator of antibiotic order in year before BMI measurement, one to capture cumulative exposure i.e. number of antibiotic orders before BMI measurement categorised as 0, 1, 2-3, 4-6 and 7+, and one to capture the cumulative number of antibiotic orders before the prior BMI. Interaction of main effects with three age terms included in model in staged-manner | Age, sex, race/ethnicity, medical assistance |
| Gerber et al. 2016^1^ (30) | Primary analysis (exposure at <6 months): 19,429/38,522 (50.0)  Secondary analysis (exposure at <24 months): 13,926/27,778 (50.0) | <6 months:  5,287/38,522 (13.7)  <24 months:  18,809/38,522 (48.8) | Not reported | Difference in rate of weight gain associated with antibiotic exposure | Singleton (main) analysis: longitudinal rate regression models, modelled using natural cubic splines (with knots at 500 and 1100 days for the primary exposure, and 1100 days for the secondary exposure)  Twin analysis: mixed effects linear regression models | Singleton analysis: adjustments made for both mean-level covariates (sex, birth weight, race, Medicaid insurance status, number of siblings, birth year, baseline length, primary care site) and rate-level covariates (as above, excluding primary care site)  Twin analysis: sex, birth weight, baseline length |
| Li et al. 2016 (31) | 134,043/260,556 (51.4) | <12 months:  138,417/260,556 (53.1) | Not reported | Adjusted OR for obesity | Singleton (main) analysis: standard mixed-effects logistic regression for repeated measurements, estimated with generalised estimating equations, using propensity score method to verify thoroughness of adjustment for confounding  Twin analysis: conditional logistic regression, with outcome defined as ever being obese | Infection type and number of episodes (diagnoses more than 2 weeks apart), maternal age and race/ethnicity, pre-pregnancy BMI, preterm delivery, low birth weight, maternal antibiotic use and infections during pregnancy, maternal education, marital status, smoking during pregnancy, mode of delivery, timing of initial prenatal care, gestational or pre-existing diabetes, breastfeeding, infant sex. |
| Scott et al. 2016 (32) | 11,094/21,714 (51.1) | <24 months  14,870/21,714 (68.5) | Obesity: 1306/21,714 (6.0) | Adjusted OR for obesity at 4 years | Logistic regression for categorical obesity risk | Maternal obesity, maternal diabetes, mode of delivery, socioeconomic status, year and country of birth, urban dwelling, and sibling obesity |
| Mbakwa et al. 2016 (33) | 490/979 (50.1) | ≤10 years  613/979 (62.6) | Not reported | Continuous: adjusted difference in sex-specific BMI-for-age z-score  Binary: adjusted OR for overweight | Generalised estimating equations with an autoregressive correlation structure | KOALA study recruitment group, household size, maternal education, maternal pre-pregnancy weight, maternal pregnancy weight gain, smoking during pregnancy, gestational diabetes, gestational hypertension, mode and place of delivery, sex, birth weight, gestational age, duration of breastfeeding, child’s dietary intake and physical activity |
| Rogawski et al. 2017 (34) | 982/1954 (50.3) | <6 months:  1284 (65.7) | Not reported | Adjusted difference in WAZ | Generalised estimating equations (multivariable linear regression) with robust variance to account for correlation of anthropometric measurements within children across time points | Site, child’s sex, enrolment weight-for-age, WAMI (water, assets maternal education and income) score, household crowding, maternal height, maternal education, characteristics of child’s first 6 months (percent days exclusively breast-fed, number of diarrhoea episodes, days with fever or vomiting or respiratory illnesses, presence of acute lower respiratory infection or bloody stools and hospitalisation) |
| BMI: body mass index; zBMI: BMI z-score; HAZ: height-for-age z-score; WAZ: weight-for-age z-score; WHZ: weight-for-height z-score; OR: Odds ratio.  ^1^% male participants and exposure and outcome prevalence reported for main analysis involving singleton births. | | | | | | |

| **Supplementary Table S4. Association between antibiotic exposure in specific age windows (months) and measures of child body mass.** | | | | | | | | | | | | | | | | | | | | | | | |
| --- | --- | --- | --- | --- | --- | --- | --- | --- | --- | --- | --- | --- | --- | --- | --- | --- | --- | --- | --- | --- | --- | --- | --- |
| **Study** | | **Summary measure** | | | | | **Group or subgroup (if relevant)** | | | **Age at outcome** | | **Association estimates for specific exposure windows (95% confidence interval or *P* value)** | | | | | | | | | | |  |
|  | | |  | | |  | | | |  | | | **<6 months** | | | **6-11 months** | | **12-17 months** | | | **18-23 months** | | |
| Saari et al. (23) | | | Adjusted odds ratio for overweight | | | | Boys | | | ≥24 months | | | 1.34 (1.06 - 1.66)* | | | 1.26 (1.02 - 1.45)* | | 1.18 (0.88 - 1.32) | | | 1.25 (0.99 - 1.61) | | |
|  |  |  |  |  |  |  | Girls | | |  | | | 1.16 (0.87 - 1.56) | | | 1.14 (0.92 - 1.42) | | 1.08 (0.85 - 1.38) | | | 1.15 (0.87 - 1.52) | | |
|  | |  | | | | |  | |  | | **<6 months** | | | | **6-14 months** | | | **15-23 months** | | | | | |
| Trasande et al. (25) | | | Adjusted difference (β) in zWHZ‡ | | | |  | | | 6 weeks | | | 0.006 (*P* = 0.778) | | | -0.039 (*P* = 0.075) | | -0.030 (*P* = 0.188) | | | | | |
|  |  |  |  |  |  |  |  | | | 10 months | | | 0.105 (*P* < 0.001)* | | | 0.040 (*P* = 0.076) | | 0.026 (*P* = 0.268) | | | | | |
|  |  |  |  |  |  |  |  | | | 20 months | | | 0.083 (*P* < 0.001)* | | | 0.023 (*P* = 0.322) | | 0.035 (*P* = 0.144) | | | | | |
|  |  |  |  | | | |  | | |  | | |  | | |  | |  | | | | | |
|  |  |  | Adjusted difference (β) in zBMI‡ | | | |  | | | 38 months | | | 0.067 (*P* = 0.009)* | | | 0.023 (*P* = 0.340) | | 0.018 (*P* = 0.463) | | | | | |
|  |  |  |  |  |  |  |  | | | 7 years | | | 0.035 (*P* = 0.183) | | | 0.022 (*P* = 0.364) | | 0.049 (*P* = 0.050) | | | | | |
|  |  |  |  | | | |  | | |  | | |  | | |  | |  | | | | | |
|  |  |  | Adjusted odds ratio for overweight^¥^ | | | |  | | | 38 months | | | 1.22 (1.01 - 1.47)* | | | 1.04 (0.86 - 1.22) | | 1.24 (0.95 - 1.38) | | | | | |
|  |  |  |  |  |  |  |  | | | 7 years | | | 1.03 (0.81 - 1.40) | | | 0.95 (0.79 - 1.18) | | 1.02 (0.87 - 1.35) | | | | | |
|  |  |  | Adjusted odds ratio for obesity^¥^ | | | |  | | | 38 months | | | 1.23 (0.96 - 1.60) | | | 1.11 (0.87 - 1.41) | | 1.20 (0.94 - 1.54) | | | | | |
|  |  |  |  |  |  |  |  | | | 7 years | | | 1.06 (0.81 - 1.36) | | | 1.05 (0.81 - 1.36) | | 0.99 (0.78 - 1.26) | | | | | |
|  |  | | |  | | | |  | | | | | | **<6 months** | | | **6-11 months** | | **12-17 months** | | | **18-23 months** | |
| Bailey et al. (26) | Adjusted hazard ratio for obesity† | | |  | | | | 24 to 59 months | | | | | | 1.07 (0.99 - 1.16) | | | 1.03 (0.6 - 1.08) | | 1.04 (0.6 - 1.11) | | | 1.03 (0.4 - 1.12) | |
|  | |  | | |  | | | |  | | **<3 months** | | | | **3-<6 months** | | | **6-<12 months** | | | | | |
| Azad et al. (27) | | | Adjusted odds ratio for overweight | | | All | | | | 9 years | | | 1.34 (0.63 - 2.83) | | | 1.83 (0.96 - 3.49) | | 1.85 (1.05 - 3.26)* | | | | | |
|  |  |  |  |  |  |  | | | | 12 years | | | 3.25 (1.40 - 7.52)* | | | 1.72 (0.79 - 3.76) | | 2.85 (1.45 - 5.58)* | | | | | |
|  |  |  |  | | |  | | | |  | | |  | | |  | |  | | | | | |
|  |  |  |  | | | Boys | | | | 9 years | | | 1.48 (0.53 - 4.14) | | | 3.70 (1.54 - 8.92)* | | 1.91 (0.87 - 4.21) | | | | | |
|  |  |  |  |  |  |  | | | | 12 years | | | 6.73 (1.88 - 24.08)* | | | 5.48 (1.72 - 17.51)* | | 5.06 (1.78 - 14.42)* | | | | | |
|  |  |  |  | | |  | | | |  | | |  | | |  | |  | | | | | |
|  |  |  |  | | | Girls | | | | 9 years | | | 1.28 (0.40 - 4.13) | | | 0.60 (0.20 - 1.82) | | 1.66 (0.67 - 4.10) | | | | | |
|  |  |  |  |  |  |  | | | | 12 years | | | 2.01 (0.57 - 7.12) | | | 0.36 (0.09 - 1.47) | | 1.48 (0.52 - 4.20) | | | | | |
|  | | |  | | |  | | | |  | | | **<6 months** | | | **<24 months** | |  | | | | | |
| Gerber et al. (30) | | | Adjusted difference (%) in rate of weight gain | | |  | | | | 2 to 5 years | | | 0.7 (-0.1 - 1.5) | | | 2.1 (0.8 - 3.3)* | |  | | | | | |
|  | | |  | | |  | | | |  | | | **<6 months** | | | **6-12 months** | |  | | | | | |
| Li et al. (31) | | | Adjusted odds ratio for obesity | | |  | | | | 2 to 18 years | | | 1.06 (1.01 - 1.10)* | | | 0.98 (0.95 - 1.01) | |  | | | | | |
|  | | |  | | |  | | | |  | | | **≤6 months** | | | **>6-12 months** | | **>12-24 months** | | | | | |
| Scott et al. (32) | | | Adjusted odds ratio for obesity | | |  | | | | 4 years | | | 1.33 (1.13 - 1.57)* | | | 1.27 (1.09 - 1.47)* | | 1.07 (0.91 - 1.26) | | | | | |
|  | | |  | | |  | | | |  | | | **≤6 months** | | | **>6-12 months** | | **>12-24 months** | | **24+ months** | | | |
| Mbakwa et al. (33) | | | Adjusted difference (β) in zBMI  Adjusted odds ratio for overweight | | |  | | | | ≤10 years  ≤10 years | | | 1 course: 0.14 (-0.05 - 0.33)  2+ courses: 0.23 (-0.21 - 0.66)  1 course: 1.21 (0.78 - 1.87)  2+ courses: 1.19 (0.38 - 3.74) | | | 1 course: 0.03 (-0.13 - 0.20)  2+ courses: 0.14 (-0.65 - 0.37)  1 course: 1.40 (0.81 - 2.42)  2+ courses: 0.83 (0.26 - 2.67) | | 1 course: 0.01 (-0.12 - 0.13)  2+ courses: 0.22 (-0.02 - 0.46)  1 course: 0.96 (0.63 - 1.46)  2+ courses: 1.14 (0.61 - 2.13) | | 1 course: 0.003 (-0.11 - 0.12)  2+ courses: 0.03 (-0.08 - 0.14)  1 course: 1.21 (0.87 - 1.70)  2+ courses: 1.02 (0.73 - 1.43) | | | |
| Measures of association are relative to unexposed group, and are presented with 95% confidence intervals unless stated otherwise.  ‡Two-tailed *P* values reported, as 95% confidence intervals were not provided; ^¥^Confidence intervals read off graph; †Estimates read off graph; * *P* < 0.05.  zWHZ: weight-for-height z-score; zBMI: body mass index-for-age and sex z-score.  See Supplementary Table S2 for adjustments relating to analyses in each study. | | | | | | | | | | | | | | | | | | | | | | | |

| **Supplementary Table S5. Association between number of antibiotic courses and measures of child body mass.** | | | | | | | | | | | | |  |
| --- | --- | --- | --- | --- | --- | --- | --- | --- | --- | --- | --- | --- | --- |
| **Study** | | **Summary measure and age at outcome** | **Antibiotic type** | | | **Group or subgroup (if relevant)** | | **Association estimates for specific categories of antibiotic course number (95% confidence interval)** | | | | |  |
|  |  | | |  |  | | **1** | | **2-3 or 2+** | **4+** | | | |
| Saari et al. (23) | Adjusted odds ratio for overweight at ≥24 months | | | Any | Boys | | 1.16 (0.92 - 1.48) | | 1.28 (1.04 - 1.59)* | 1.27 (1.04 - 1.55)* | | | |
|  |  |  |  |  | Girls | | 1.03 (0.81 - 1.32) | | 1.14 (0.91 - 1.42) | 1.19 (0.96 - 1.48) | | | |
|  |  |  |  |  |  | |  | |  |  | | | |
|  |  |  |  | Penicillins | Boys | | 1.13 (0.90 - 1.41) | | 1.29 (1.05 - 1.58)* | 1.35 (1.07 - 1.69)* | | | |
|  |  |  |  |  | Girls | | 1.11 (0.89 - 1.40) | | 1.19 (0.96 - 1.48) | 1.11 (0.85 - 1.44) | | | |
|  |  |  |  |  |  | |  | |  | | | | |
|  |  |  |  | Cephalosporins | Boys | | 1.39 (1.10 - 1.76)* | | 1.15 (0.84 - 1.58) | | | | |
|  |  |  |  |  | Girls | | 1.05 (0.80 - 1.38) | | 1.46 (1.03 - 2.05)* | | | | |
|  |  |  |  |  |  | |  | |  | | | | |
|  |  |  |  | Macrolides | Boys | | 1.20 (0.96 - 1.49) | | 1.47 (1.16 - 1.86)* | | | | |
|  |  |  |  |  | Girls | | 0.94 (0.94 - 1.50) | | 0.88 (0.88 - 1.55) | | |  | |
|  |  | | |  |  | | **1** | | **2** | **3** | | **4+** | |
| Bailey et al. (26) | Adjusted hazard ratio for obesity at 24 to 59 months | | | Any |  | | 1.03 (0.97 - 1.09) | | 1.04 (0.96 - 1.13) | 1.04 (0.95 - 1.14) | | 1.11 (1.02 - 1.21)* | |
|  |  |  |  | Broad spectrum† |  | | 1.08 (1.05 - 1.12)* | | 1.06 (1.01 - 1.12)* | 1.05 (0.4 - 1.16) | | 1.17 (1.06 - 1.28)* | |
|  |  |  |  | Narrow spectrum† |  | | 1.01 (0.7 - 1.05) | | 0.9 (0.2 - 1.06) | 0.9 (0.2 - 1.08) | | 1.08 (0.7 - 1.18) | |
|  |  | | |  |  | | **1-2** | | **3-4** | **4+** | |  | |
| Azad et al. (27) | Adjusted odds ratio for overweight | | | Any | All – 9 years | | 2.02 (1.15 - 3.55)* | | 1.26 (0.64 - 2.48) | 1.77 (0.87 - 3.62) – *P*(trend) = 0.47 | | | |
|  |  |  |  |  | All – 12 years | | 3.07 (1.57 - 6.00)* | | 2.12 (0.98 - 4.61) | 1.85 (0.78 - 4.37) – *P*(trend) = 0.13 | | | |
|  |  |  |  |  |  | |  | |  |  | |  | |
|  |  |  |  |  | Boys – 9 years | | 2.15 (0.99 - 4.65) | | 2.25 (0.91 - 5.57) | 2.26 (0.88 - 5.82) – *P*(trend) = 0.77 | | | |
|  |  |  |  |  | Boys – 12 years | | 5.86 (2.08 - 16.56)* | | 4.60 (1.42 - 14.90)* | 4.46 (1.30 - 15.36)* – *P*(trend) = 0.37 | | | |
|  |  |  |  |  |  | |  | |  |  | |  | |
|  |  |  |  |  | Girls – 9 years | | 1.89 (0.76 - 4.75) | | 0.46 (0.14 - 1.51) | 1.42 (0.42 - 4.78) – *P*(trend) = 0.19 | | | |
|  |  |  |  |  | Girls – 12 years | | 1.44 (0.52 - 4.03) | | 1.04 (0.31 - 3.43) | 0.68 (0.15 - 2.99) – *P*(trend) = 0.39 | | | |
|  |  | | |  |  | | **1** | | **2+** |  | |  | |
| Rogawski et al. 2015 (28) | Adjusted difference (β) in weight-for-length z-score at 6 months to 3 years | | | Any |  | | -0.11(-0.26 - 0.03) | | -0.10 (-0.24 - 0.05) |  | |  | |
|  |  | | |  |  | | **1** | | **2** | **3+** | |  | |
| Gerber et al. (30) | Adjusted difference in rate of weight gain (%) between 2 and 5 years | | | Any | Exposed <6 months  Exposed <24 months | | 1.0 (0.1 - 1.8)*  1.8 (0.4 - 3.3)* | | 0.3 (-1.5 - 2.1)  1.9 (0.3 - 3.6)* | -1.7 (-1.4 - 1.5)  2.4 (0.9 - 3.9)* | |  | |
|  |  | | |  |  | | **1-2** | | **3-5** | **5+** |  | | |
| Scott et al. (32) | Adjusted odds ratio for obesity at 4 years | | | Any | All | | 1.07 (0.93 - 1.23) | | 1.41 (1.20 - 1.65)* | 1.47 (1.19 - 1.82)* |  | | |
|  |  | | |  |  | | **1** | | **2-3** | **4+** | |  | |
| Mbakwa et al. (33) | Adjusted difference (β) in zBMI at ≤10 years  Adjusted odds ratio for overweight at ≤10 years | | | Any | All | | 0.03 (-0.11 - 0.18)  1.09 (0.76 - 1.57) | | 0.07 (-0.05 - 0.19)  1.13 (0.79 - 1.63) | 0.01 (-0.11 - 0.13)  1.03 (0.66 - 1.60) | |  | |
| Measures of association are relative to unexposed group unless stated otherwise.  zBMI – body mass index-for-age and sex z-score.  †Estimates read off graph; * *P* < 0.05.  See Supplementary Table S2 for adjustments relating to analyses in each study. | | | | | | | | | | | | |  |

| **Supplementary Table S6. Predicted weight gain associated with antibiotic exposure as a proportion of age-specific median weight among children.** | | | | | | |
| --- | --- | --- | --- | --- | --- | --- |
| **Study** | **Age** | **Predicted weight gain with exposure to antibiotics (kg)** | **Age-specific population median weight (kg)** | | **Predicted weight gain as a proportion of age-specific population median weight (%)** | |
|  |  |  | **Boys** | **Girls** | **Boys** | **Girls** |
| Rogawski et al. 2015 (boys) (28) | 24 months | 0.001 | 12.2 | 11.5 | 0.008 |  |
| Rogawski et al. 2015 (girls) (28) | 24 months | 0.032 | 12.2 | 11.5 |  | 0.278 |
| Trasande et al. 2012 (25) | 38 months | 0.09 | 14.7 | 14.2 | 0.612 | 0.634 |
| Bailey et al. 2014 (26) | 24 to 60 months | 0.15 | 18.3 | 18.2 | 0.820 | 0.824 |
| Schwartz et al. 2016 (29) | 15 years | 1.5 | 56.3 | 52.0 | 2.665 | 2.882 |
| Age-specific population median weight was according to the WHO 2006 Standard for children up to 60 months, and the CDC 2000 reference for children aged greater than 60 months. For children aged 15 years (180 months), median weight was calculated as the average of median weight at 179.5 months and 180.5 months. | | | | | | |

**References**

1. Guerrant RL, DeBoer MD, Moore SR, Scharf RJ, Lima AAM. The impoverished gut—a triple burden of diarrhoea, stunting and chronic disease. Nature reviews Gastroenterology & hepatology. 2013;10(4):220-9.

2. Victora CG, Adair L, Fall C, Hallal PC, Martorell R, Richter L, et al. Maternal and child undernutrition: consequences for adult health and human capital. Lancet. 2008;371(9609):340-57.

3. Margolis R. Childhood Morbidity and Health in Early Adulthood: Life course linkages in a high morbidity context. Advances in life course research. 2010;15(4):132-46.

4. Kuhle S, Tong OS, Woolcott CG. Association between caesarean section and childhood obesity: a systematic review and meta-analysis. Obes Rev. 2015;16(4):295-303.

5. Kero J, Gissler M, Gronlund MM, Kero P, Koskinen P, Hemminki E, et al. Mode of delivery and asthma -- is there a connection? Pediatr Res. 2002;52(1):6-11.

6. Roduit C, Scholtens S, de Jongste JC, Wijga AH, Gerritsen J, Postma DS, et al. Asthma at 8 years of age in children born by caesarean section. Thorax. 2009;64(2):107-13.

7. Rautava S, Walker WA. Academy of Breastfeeding Medicine founder's lecture 2008: breastfeeding--an extrauterine link between mother and child. Breastfeeding medicine : the official journal of the Academy of Breastfeeding Medicine. 2009;4(1):3-10.

8. Groer MW, Gregory KE, Louis-Jacques A, Thibeau S, Walker WA. The very low birth weight infant microbiome and childhood health. Birth defects research Part C, Embryo today : reviews. 2015;105(4):252-64.

9. Butel MJ, Suau A, Campeotto F, Magne F, Aires J, Ferraris L, et al. Conditions of bifidobacterial colonization in preterm infants: a prospective analysis. J Pediatr Gastroenterol Nutr. 2007;44(5):577-82.

10. Burke H, Leonardi-Bee J, Hashim A, Pine-Abata H, Chen Y, Cook DG, et al. Prenatal and passive smoke exposure and incidence of asthma and wheeze: systematic review and meta-analysis. Pediatrics. 2012;129(4):735-44.

11. Oken E, Levitan EB, Gillman MW. Maternal smoking during pregnancy and child overweight: systematic review and meta-analysis. Int J Obes (Lond). 2008;32(2):201-10.

12. Wang Y, Lim H. The global childhood obesity epidemic and the association between socio-economic status and childhood obesity. International review of psychiatry (Abingdon, England). 2012;24(3):176-88.

13. Gong T, Lundholm C, Rejnö G, Mood C, Långström N, Almqvist C. Parental Socioeconomic Status, Childhood Asthma and Medication Use – A Population-Based Study. PLOS ONE. 2014;9(9):e106579.

14. Blais L, Beauchesne M-F, Lévesque S. Socioeconomic status and medication prescription patterns in pediatric asthma in Canada. Journal of Adolescent Health. 2006;38(5):607.e9-.e16.

15. Parikka S, Maki P, Levalahti E, Lehtinen-Jacks S, Martelin T, Laatikainen T. Associations between parental BMI, socioeconomic factors, family structure and overweight in Finnish children: a path model approach. BMC Pub Health. 2015;15:271.

16. Kalliomaki M, Collado MC, Salminen S, Isolauri E. Early differences in fecal microbiota composition in children may predict overweight. Am J Clin Nutr. 2008;87(3):534-8.

17. Collado MC, Isolauri E, Laitinen K, Salminen S. Distinct composition of gut microbiota during pregnancy in overweight and normal-weight women. Am J Clin Nutr. 2008;88(4):894-9.

18. Han Z, Mulla S, Beyene J, Liao G, McDonald SD. Maternal underweight and the risk of preterm birth and low birth weight: a systematic review and meta-analyses. Int J Epidemiol. 2011;40(1):65-101.

19. Foliaki S, Pearce N, Bjorksten B, Mallol J, Montefort S, von Mutius E. Antibiotic use in infancy and symptoms of asthma, rhinoconjunctivitis, and eczema in children 6 and 7 years old: International Study of Asthma and Allergies in Childhood Phase III. The Journal of allergy and clinical immunology. 2009;124(5):982-9.

20. Reinehr T. Lifestyle intervention in childhood obesity: changes and challenges. Nat Rev Endocrinol. 2013;9(10):607-14.

21. Pape K, Ryttergaard L, Rotevatn TA, Nielsen BJ, Torp-Pedersen C, Overgaard C, et al. Leisure-Time Physical Activity and the Risk of Suspected Bacterial Infections. Medicine and science in sports and exercise. 2016;48(9):1737-44.

22. Ajslev TA, Andersen CS, Gamborg M, Sorensen TI, Jess T. Childhood overweight after establishment of the gut microbiota: the role of delivery mode, pre-pregnancy weight and early administration of antibiotics. Int J Obes (Lond). 2011;35(4):522-9.

23. Saari A, Virta LJ, Sankilampi U, Dunkel L, Saxen H. Antibiotic exposure in infancy and risk of being overweight in the first 24 months of life. Pediatrics. 2015;135(4):617-26.

24. Murphy R, Stewart AW, Braithwaite I, Beasley R, Hancox RJ, Mitchell EA. Antibiotic treatment during infancy and increased body mass index in boys: an international cross-sectional study. Int J Obes (Lond). 2014;38(8):1115-9.

25. Trasande L, Blustein J, Liu M, Corwin E, Cox LM, Blaser MJ. Infant antibiotic exposures and early-life body mass. Int J Obes (Lond). 2013;37(1):16-23.

26. Bailey LC, Forrest CB, Zhang P, Richards TM, Livshits A, DeRusso PA. Association of antibiotics in infancy with early childhood obesity. JAMA Pediatr. 2014;168(11):1063-9.

27. Azad MB, Bridgman SL, Becker AB, Kozyrskyj AL. Infant antibiotic exposure and the development of childhood overweight and central adiposity. Int J Obes (Lond). 2014;38(10):1290-8.

28. Rogawski ET, Westreich DJ, Adair LS, Becker-Dreps S, Sandler RS, Sarkar R, et al. Early Life Antibiotic Exposure Is Not Associated with Growth in Young Children of Vellore, India. J Pediatr. 2015;167(5):1096-102.e3.

29. Schwartz BS, Pollak J, Bailey-Davis L, Hirsch AG, Cosgrove SE, Nau C, et al. Antibiotic use and childhood body mass index trajectory. Int J Obes (Lond). 2016;40(4):615-21.

30. Gerber JS, Bryan M, Ross RK, et al. Antibiotic exposure during the first 6 months of life and weight gain during childhood. JAMA. 2016;315(12):1258-65.

31. Li D-K, Chen H, Ferber J, Odouli R. Infection and antibiotic use in infancy and risk of childhood obesity: a longitudinal birth cohort study. The Lancet Diabetes & Endocrinology. 2016.

32. Scott FI, Horton DB, Mamtani R, Haynes K, Goldberg DS, Lee DY, et al. Administration of Antibiotics to Children Before Age 2 Years Increases Risk for Childhood Obesity. Gastroenterology. 2016;151(1):120-9.e5.

33. Mbakwa CA, Scheres L, Penders J, Mommers M, Thijs C, Arts ICW. Early Life Antibiotic Exposure and Weight Development in Children. J Pediatr. 2016;176(Supplement C):105-13.e2.

34. Rogawski ET, Platts-Mills JA, Seidman JC, John S, Mahfuz M, Ulak M, et al. Early Antibiotic Exposure in Low-resource Settings Is Associated With Increased Weight in the First Two Years of Life. J Pediatr Gastroenterol Nutr. 2017;65(3):350-6.
